# Passionfruit Genomic Database (PGD): a comprehensive resource for passionfruit genomics

**DOI:** 10.1186/s12864-024-10069-9

**Published:** 2024-02-08

**Authors:** Chaowei Yu, Peng Wang, Shengjie Zhang, Jindian Liu, Yingyin Cheng, Songbai Zhang, Zujian Wu

**Affiliations:** 1 MARA Key Laboratory of Sustainable Crop Production in the Middle Reaches of the Yangtze River (Co-Construction By Ministry and Province), Yangtze University, Jingzhou, 434025 China; 2 Institute of Plant Virology, Fujian Agriculture and Forestry University, Fuzhou, 350002 China

**Keywords:** Passionfruit genomic database, *Passiflora edulis*, Functional genomics, Transcriptomics, Web server

## Abstract

Passionfruit (*Passiflora edulis*) is a significant fruit crop in the commercial sector, owing to its high nutritional and medicinal value. The advent of high-throughput genomics sequencing technology has led to the publication of a vast amount of passionfruit omics data, encompassing complete genome sequences and transcriptome data under diverse stress conditions. To facilitate the efficient integration, storage, and analysis of these large-scale datasets, and to enable researchers to effectively utilize these omics data, we developed the first passionfruit genome database (PGD). The PGD platform comprises a diverse range of functional modules, including a genome browser, search function, heatmap, gene expression patterns, various tools, sequence alignment, and batch download, thereby providing a user-friendly interface. Additionally, supplementary practical tools have been developed for the PGD, such as gene family analysis tools, gene ontology (GO) terms, a pathway enrichment analysis, and other data analysis and mining tools, which enhance the data’s utilization value. By leveraging the database’s robust scalability, the intention is to continue to collect and integrate passionfruit omics data in the PGD, providing comprehensive and in-depth support for passionfruit research. The PGD is freely accessible via http://passionfruit.com.cn.

## Background

*Passiflora* is the largest and most widely distributed genus in the Passifloraceae family, comprising about 500 species [1]. The genus contains many species with edible fruits, but only a few have commercial value. Passionfruit, a native plant of South America, is widely cultivated around the world because of its economic value [2]. The fruit is round or oval, with a tough outer rind and juicy, aromatic pulp inside. It is commonly used in juices, desserts, and as a flavoring for other foods [3–5]. In addition to their edible fruits, some species of *Passiflora* have medicinal properties and are used in traditional herbal medicine [6, 7]. Many region-specific traditional cultivars are widely promoted and cultivated [4, 8] but only a few varieties are recognized in the market, including the purple *Passiflora edulis* Sims (purple passionfruit) and the yellow form *Passiflora edulis* f. *flavicarpa* Degener (yellow passionfruit) [3]. Although these two passionfruit cultivars have notably market value and a remarkable survival capacity, like other passionfruit cultivars, their yield and quality are easily affected by biotic and abiotic factors in the growing environment, resulting in a significant decline in planting benefits. Therefore, it is necessary to conduct further research on this species, master the basic knowledge needed to improve yields and stress resistance, and ensure its safe agricultural production.

The lack of publicly available tools to support fundamental research in *Passiflora* species can be attributed to factors such as their large, complex genomes. Despite these challenges, multiple data sets covering various aspects of *Passiflora* genetics and genomics have been published over the last decade. Santos et al. [8] have constructed and characterized the first large-insert bacterial artificial chromosome (BAC) library of *Passiflora edulis*. In 2021, two high-quality purple *Passiflora edulis* genome sequences were reported, with assembled sequences of 1.28 Gb [9] and 1.31 Gb [10], respectively. These genome sequences predicted 39,309 and 23,171 protein-coding genes, respectively, and were both assembled onto nine chromosomes.

Although there are many public data sets that contain the data mentioned above, these data are only present in central public databases, which was not suitable for our genomic analysis of passionfruit. To harness all the genomic and transcriptomic data available on passionfruit and foster research on this species, a dedicated repository is necessary. Therefore, we established a genomic database, the passionfruit genome database (PGD), which was the first integrative functional database that focuses on passionfruit species. The PGD integrates multiple types of omics data of passionfruit, and has a set of online tools for commonly used bioinformatics analyses. To ensure that we can update data or allow users to add data for analysis at any time, we have integrated various visualization platforms and tools, including JBrowse. In summary, we have established a comprehensive passionfruit database with integrative functionality.

The PGD database includes functions such as browsing, retrieval, online analysis, and genomic visualization. This article describes the design, functions, and applications of the PGD portal, and briefly outlines existing technologies for developing genomic portals, as well as the potential for enriching data and enhancing analytical capabilities in the future. Finally, we presented a case study illustrating the practical application of the portal and discussing its potential to promote further genomic research on passionfruit.

## Construction and content 

### Genome data sources and annotation

We downloaded the genome sequence of purple passionfruit (*Passiflora edulis* Sims) (PRJCA004251) from the China National Center for Bioinformation (CNCB) (https://www.cncb.ac.cn/). For yellow passionfruit (*Passiflora edulis* f. *flavicarpa* Degener.), we performed whole genome sequencing and obtained a high-quality genome assembly sequence at the chromosome level (CNCB, PRJCA020234 or http://passionfruit.com.cn/downloads_data.html).

**Fig. 1:**
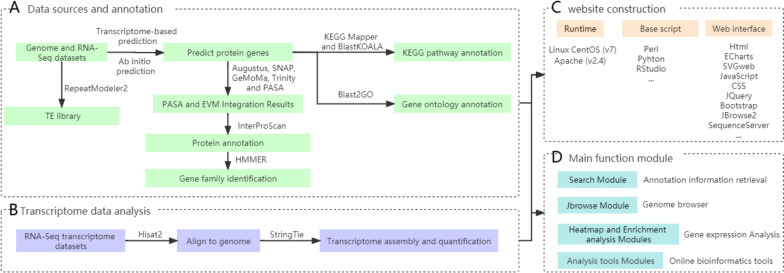
Constructing PGD via prior pipeline. **A** Genome data sources and annotation. **B** Transcriptome data analysis (**C**) Data integration and website construction. **D** Main function module

After obtaining the whole genome sequence of passionfruit, we used a custom program for genome (re)annotation. RepeatModeler2 [11] (v2.0.1) was used for ab initio prediction and homology-based annotation of transposable elements (TEs) in both passionfruit genomes, and a TEs library for this species was also established. To predict protein-coding genes, three complementary strategies were employed, including ab initio prediction (Augustus [12] (v3.1.0), SNAP [13] (v4.0)), homology-based prediction (GeMoMa [14] (v1.7)), and transcriptome-based prediction (Trinity [15] (v2.11) and PASA [16] (v2.4.1)), which were integrated using PASA and EVM [17] (v1.1.1) software. Additionally, the protein domain and motif annotation were performed using the InterProScan [18] (v5.39–77.0) software to search multiple databases such as SMART [19], InterPro [20], PROSITE [21]. Finally, the HMMER [22] software was used to identify gene families. The Kyoto Encyclopedia of Genes and Genomes (KEGG) Mapper [23–26], BlastKOALA (https://www.kegg.jp/blastkoala/), and Blast2GO [27] (v2.7.2) were used for KEGG pathway and gene ontology (GO) annotation, respectively. All annotation results were stored in a specific format in databases for easy accessibility (Fig. 1A).

### Transcriptome data analysis

To obtain a better understanding of the gene expression patterns, we obtained 96 RNA-Seq transcriptome datasets from seven different treatment conditions, including 24 tissues, six heat-treated samples, six cold-treated samples, six salt-treated samples, six drought-treated samples, 30 hormone-treated samples, and 18 virus-treated samples. After filtering the raw data with fastqc, high-quality sequencing data were obtained (CNCB,PRJCA020235). We used Hisat2 [28] (v2.0.4) software to map clean RNA-seq data to the corresponding reference genome, and conducted a transcriptome assembly and quantification using StringTie [29] (v1.3.3) software and the fragments per kilobase of gene/transcript model per Million mapped fragments (FPKM) method. Finally, we reconstructed a single expression matrix file based on the common gene ID of the 96 transcriptome expression datasets for further analysis (Fig. 1B).

### Data integration and website construction

The PGD consists of a front-end web interface, a back-end application server, and a suite of Perl, R, and Python scripts for data processing, analysis, and visualization. The web server runs on a Linux server based on CentOS (v7) and Apache (v2.4). To improve the user experience, the PGD uses technologies such as JavaScript and CSS libraries, JQuery, and Bootstrap to enhance the website interface. The PGD also uses custom Perl scripts to process user interaction data and extract feature data. Additionally, the PGD integrates several open-source plugins such as ECharts (http://echarts.baidu.com/) and SVGweb (https://code.google.com/p/svgweb/) to dynamically display data. Furthermore, the PGD includes JBrowse, an open-source and full-featured genome browser based on JavaScript and HTML5. The BLAST online search tool was built using SequenceServer (v2.0) software (http://www.sequenceserver.com/).Finally, we have uploaded the web code for developing PGD to GitHub. (https://github.com/yuchaowei2023/Passionfruit_Genomic_Database). In order to enrich the usability of the database, we developed an online toolkit for researchers to perform a customized gene family analysis (Fig. 1C, D).

## Utility and discussion

### Essential modules and interface of PGD

The PGD database consists of seven main functional modules: Search, Browse, Heatmap, Enrichment Analysis, Tools, Download, and User Guide. These functional modules can be used separately or work collaboratively as a whole.

The PGD interface is divided into several sections: quick data search, navigation bar, PGD introduction, and a column for commonly used gene family analysis tools. The quick data search module is located at the top of the page. Underneath it there is a navigation bar comprising five tabs: Home, Search, Genome Browser, Gene Expression Analysis, Analysis Tools, User Guide, and Data Download. In the middle of the page there is a PGD summary and seven frequently used tools. At the bottom of the homepage, there are four user-friendly links that lead to corresponding frequently used functional modules.

### Search module of PGD

The PGD search module provides two search category options, “Quick Search” and “Annotation Search.”

**Fig. 2:**
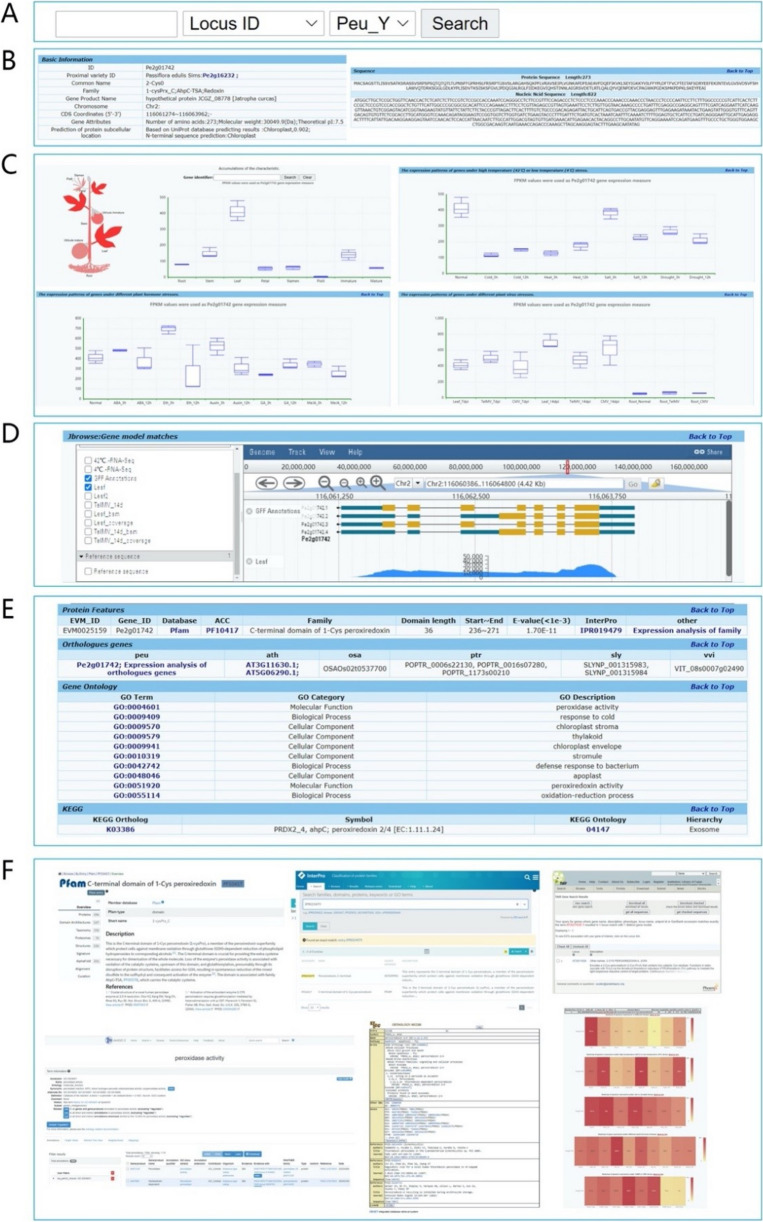
The page of gene detailed information introduction. **A** “Quick search” using keywords or annotation information. **B** Gene basic information. **C** Gene expression pattern diagram. **D** Gene information in JBrowse. **E**, **F** KEGG pathway and GO functional annotation of gene

Users can quickly search the database by clicking the “Search” button at the top of the page (Fig. 2A). The search information includes species selection, locus ID, keywords, or other annotation IDs (such as KEGG pathway, GO terms, and Pfam ID) (Fig. 2A). The locus ID search results include detailed basic gene information (Fig. 2B), such as chromosome location, protein physicochemical properties, characteristic structural domains or motifs, homologous genes, coding sequence (CDS), and peptide sequence. Expression data from different tissues and biotic/abiotic stresses can be displayed as plant sketch heatmaps or boxplots (Fig. 2C). To facilitate the display of gene location and gene structure information in the genome, a small genome browser JBrowse2 is embedded into the gene information introduction page (Fig. 2D). The KEGG pathway and GO functional annotation results are presented as a table (Fig. 2E). Links are provided in the corresponding entries to the source pages of the original annotation databases, such as KEGG, GO, or InterPro ID, to obtain more comprehensive information (Fig. 2F).

The drop-down menu of “Annotation Search” provides a list of annotation dataset search links: Gene Locus, Motif/Domain, KEGG, GO, SSR (Simple Sequence Repeat) markers and non-coding RNA annotation data. Each search page can filter data by the two reference species (yellow passionfruit and purple passionfruit), annotation classification, annotation name, and list the gene symbols and total number that meet the criteria in tabular form. To enable users to view detailed gene information, users can click on the gene symbol hyperlink to jump to the gene information interface. Similarly, other result entries can also hyperlink to the original annotation database source page.

### Jbrowse module of PGD

**Fig. 3:**
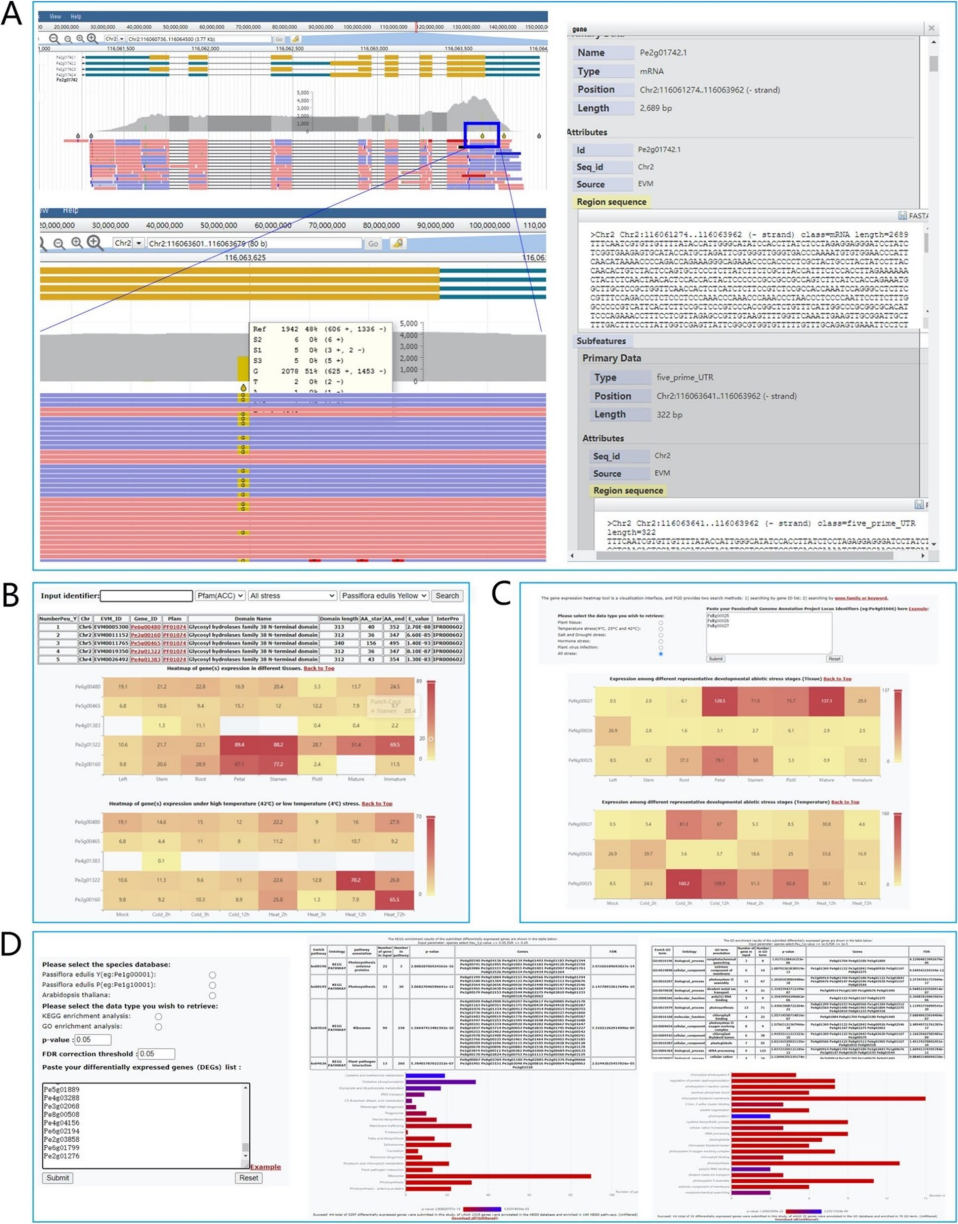
The “Genome Browser,” “Heatmap,” and “KEGG and GO Enrichment” modules of PGD. **A** The “Genome Browser” module provides genomic data, including gene structure and location, genome and transcript sequence, and SNPs loci. **B**, **C** A dynamic, editable heatmap retrieved by the user. **D** The “KEGG and GO Enrichment” shows enriched KEGG and GO terms within a query gene list

JBrowse is a widely used interactive genome visualization browser for displaying genome data resources, including genome sequences, gene structures, protein-coding gene annotations, single nucleotide polymorphisms (SNPs) site information, and expression profiles. We integrated the JBrowse2 tool into the PGD and imported all publicly available passionfruit RNA-Seq, genome data, and annotation data (General feature format files) into JBrowse2 (Fig. 3A). To improve Jbrowse’s query function, we added an ElasticSearch module. Users can not only query by region range but also use keywords to accurately search for genes of interest. In addition, users can upload and analyze their own omics data through tracks and download the corresponding reports.

### Heatmap and enrichment analysis modules of PGD

We developed a visualization interface for a gene expression heatmap, providing two search methods: searching by gene family or keyword (Fig. 3B), or by a list of gene IDs (Fig. 3C).

Next-generation sequencing (NGS) is one of the most effective methods for studying biological functions. The NGS method can generate and fully analyze many differentially expressed genes (DEGs), which is a prerequisite for studying the potential mechanisms of biological processes. A functional enrichment analysis (e.g., GO terms and KEGG pathways) is a commonly used method to identify major biological functions from DEG datasets. Therefore, we developed two functional modules based on Perl and R scripts to perform a GO functional enrichment analysis and KEGG pathway enrichment analysis on DEG datasets provided by users (Fig. 3D). These modules can effectively identify significantly enriched GO terms and KEGG pathways from DEGs, providing important information for further research on biological processes.

### Analysis tools modules of PGD

The PGD offers a very useful suite of analysis and visualization bioinformatics tools to explore and analyze genomic data.

#### BLAST

The “BLAST” tool permits the user to paste queries relating to one or more sequences in the input box, drag and drop FASTA format files, and then select BLAST parameters, including preformatted database, programs (blastn, blastp, blastx, tblastn, and tblstx), and an E-value cutoff (Figure S1A).

#### Gene family search

In the “Gene Family Search” tool, users can search by Pfam ID and set filtering parameters such as genome database, control report type, and threshold screening. On the output page, users can not only download the filtered gene family list, but also truncate domain sequences based on the threshold and draw sequence polymorphism diagrams (Figure S1B).

#### Gene structure analysis

The gene structure analysis tool can not only obtain gene structure information in batches based on the user-input gene list, but also draw gene structure maps online (Figure S1C).

#### Protein physicochemical properties online analysis

To support the batch prediction of protein physicochemical properties, we developed an online tool for predicting protein physicochemical properties. The prediction results are presented in table form, including various properties related to protein properties, such as protein length (AA), molecular weight (MW), theoretical isoelectric point (Pi), and protein hydrophilicity grand average (GRAVY) (Figure S1D).

#### The K_a_/K_s_ calculation for homologous genes

To facilitate the calculation of the K_a_/K_s_ ratio for homologous genes, we integrated a K_a_/K_s_ calculator into the PGD. Users only need to provide a list of homologous genes for a K_a_/K_s_ calculation, and the results are provided in table and K_a_/K_s_ scale distribution formats (Figure S1E).

#### A case study for the application of the PGD

The lipoxygenase (LOX) protein gene family of passionfruit was analyzed using the PGD platform (Figure S2). LOX is a multifunctional enzyme in plants, characterized by its non-heme iron-containing dioxygenases. Its functions include participation in seed development, tissue development, and fruit flavor formation, as well as playing a crucial role in the plant response to stressors such as pathogen infection, drought, and salt stress. Previous studies have demonstrated the significance of LOX in the synthesis of volatile compounds in fruits, making the analysis of the LOX gene family essential for understanding the rich and unique aroma of passionfruit. The “Protein family prediction” module was employed to search for candidate genes in the genome libraries of purple and yellow passionfruit using the “lipoxigenase” domain “PF00305” in Pfam as an input and a full-length alignment threshold E-value of 1e-5. Subsequently, the results were subjected to the “Physicochemical properties of protein analysis tool” and “Gene structure analysis tool” to filter and screen them (Figure S2B), resulting in the identification of 17 and 12 LOX genes in yellow and purple passionfruit, respectively. The K_a_/K_s_ analysis of homologous LOX genes in both passionfruit varieties revealed a K_a_/K_s_ ratio that was predominantly concentrated between 0 and 0.15 (Figure S2C), it was observed that the LOX genes underwent gene purification selection in both varieties. To conduct a phylogenetic tree analysis of the passionfruit LOX gene, we employed the same methodology on the PGD platform to identify LOX in the complete genome of *Arabidopsis thaliana*. Subsequently, utilizing the LOX protein sequences of passionfruit and *A. thaliana*, we utilized the mega software to construct a maximum likelihood phylogenetic tree (Figure S2A). The findings of the analysis revealed that the LOX protein was classified into two subfamilies, namely 9-LOXs and 13-LOXs. Furthermore, we also analyzed the expression pattern of the LOX gene in yellow passion fruit (Figure S2D). The PGD platform's forecast of LOX in purple passionfruit was aligned with the findings of the LOX gene family identification in purple passionfruit by Huang et al. [30]. underscoring the PGD platform's immense potential in gene family analysis.

## Conclusions

The progress of passionfruit genomics research is still in its early stages compared to other important economic crops. However, recent studies have reported a wealth of research findings on the gene families of passion fruit. By utilizing high-throughput sequencing technology and bioinformatics tools, they have successfully identified multiple gene families in the passionfruit genome. Through the study of these gene families, researchers have begun to understand the genomic characteristics and functions of passionfruit. For example, they have discovered that some disease resist.

ance gene families play an important protective role in passionfruit, helping it resist various biological and abiotic stresses [30–35]. Additionally, some growth and development regulation gene families have been found to be closely related to the fruit growth and ripening process of passionfruit [30, 35]. These research findings contribute to a better understanding of the biological characteristics of passionfruit and provide a theoretical basis for further genetic improvement and cultivation. Although passionfruit genomics research is still in its early stages, encouraging progress has already been made.

To enhance the accessibility, analysis, and visualization of passionfruit genomics data, the PGD web portal was developed. As a sustainable solution, the PGD constructed a comprehensive central portal for passionfruit genomics, which will amalgamate diverse omics data and create a suite of omics data mining tools. To date, the PGD has amassed a range of passionfruit omics data, comprising genome assembly sequences, protein-coding gene structural and functional annotation, non-coding gene annotation, homologous gene pair data, and gene expression profiles. Furthermore, the PGD website offers a plethora of interactive analysis tools, enabling basic and batch queries, download options, BLAST, use of a genome browser, screening and analysis of gene families, determination of the physicochemical properties of proteins, gene structure analysis, conserved protein motif analysis, KEGG pathway and GO term enrichment analysis, and calculations of the substitution ratios (K_a_/K_s_) of orthologous gene-pairs. In conclusion, the PGD offers a diverse range of tools to facilitate the querying, analysis, and visualization of molecular interactions. As a new genomic dataset, with the potential to include genomes and transcriptomes that become publicly accessible for passionfruit species, the PGD will be regularly updated. It is envisaged that the PGD will serve as a valuable platform for conducting functional genomics research and molecular breeding endeavors in passionfruit.

## Data Availability

Data generated or analysed during this study are already publicly available, The purple passionfruit (*Passiflora edulis* Sims) genome data can be found at the following website: https://ngdc.cncb.ac.cn/search/?dbId=&q=PRJCA004251. In addition, other data can be downloaded from the PGD: http://passionfruit.com.cn/Downloads.html.
